# Cellular angiofibroma of the vulva: case report with clinicopathological and immunohistochemistry study

**DOI:** 10.1590/S1516-31802005000500010

**Published:** 2005-09-01

**Authors:** Adilha Misson Rua Micheletti, Ana Cristina Araújo Lemos da Silva, Antonio Geraldo Nascimento, Cléber Sérgio Da Silva, Eddie Fernando Candido Murta, Sheila Jorge Adad

**Keywords:** Angiofibroma, Connective tissue, Mesoderm, Neoplasm, Vulva, Angiofibroma, Tecido conjuntivo, Mesênquima, Neoplasia, Vulva

## Abstract

**CONTEXT:**

Cellular angiofibroma of the vulva is a rare tumor that was first described in 1997. It occurs in middle-aged women (average age: 47 years), has small size (< 3 cm) and well-circumscribed margins.

**CASE REPORT:**

We describe a case in a 51-year-old woman whose preoperative diagnosis was confounded with Bartholin's glandular cyst. The neoplasia was well delimited and made up of three characteristic components: fusiform cells forming small fascicles, numerous blood vessels and adipose tissue interspersed between the fusiform cells. The stroma cells were positive for vimentin and negative for CD34, protein S-100, actin and desmin. The differential diagnoses for this tumor include aggressive angiomyxoma, angiomyofibroblastoma, lipoma of fusiform cells, solitary fibrous tumor, perineurioma and leiomyoma.

## RESUMO

**CONTEXTO:**

O angiofibroma celular da vulva é um tumor raro que foi inicialmente descrito em 1997. Ocorre em mulheres de meia-idade (média de idade: 47 anos), apresentar pequeno tamanho (< 3 cm) e margem bem circunscrita.

**RELATO DE CASO:**

Descrevemos um caso em mulher de 51 anos de idade cujo diagnóstico pré-operatório foi confundido com cisto de glândula de Bartholin. A neoplasia era bem delimitada e constituída por três componentes característicos: células fusiformes formando pequenos fascículos, numerosos vasos sangüíneos e tecido adiposo entremeado às células fusiformes. As células do estroma eram positivas para vimentina e negativas para CD34, proteína S-100, actina e desmina. O diagnóstico diferencial deste distinto tumor inclui angiomixoma agressivo, angiomiofibroblastoma, lipoma, tumor fibroso solitário, perineurioma, e leiomioma.

## INTRODUCTION

The mesenchymal lesions of the vulva and perineum include both benign and malignant neoplasias. Cellular angiofibroma is a rare tumor described for the first time by Nucci et al. in 1997.^1^ It consists of a tumoral mass of small size (< 3 cm) that is generally well circumscribed, and it typically arises in middle-aged patients.^2^ The differential diagnoses for this neoplasia include aggressive angiomyxoma, angiomyofibroblastoma, lipoma of fusiform cells, fibrous tumors, perineurioma and leiomyoma. This differentiation is done by means of the histological and immunohistochemical characteristics.^1^ We describe a case of cellular angiofibroma dealt with in our institution, for which the preoperative diagnosis was Bartholin's glandular cyst. To the best of our knowledge, this is the first case described in the Brazilian literature.

## CASE REPORT

The patient was a 51-year-old woman attended as an outpatient with a complaint of tumor formation in the vulva, with progressive growth detected two years earlier. The tumor was painless, and the complaint was of discomfort when seated and a localized burning sensation. Upon gynecological examination, she presented a tumor of cystic consistency, located between the right-side small and large vulvar labia, and measuring approximately 7 cm in diameter. Her obstetric history consisted of three children: two cesarean and one normal delivery. The clinical diagnosis was Bartholin's glandular cyst, and the patient underwent elective surgery. The resected material was sent for histopathological examination.

## PATHOLOGICAL AND IMMUNOHISTOCHEMICAL FINDINGS

The surgical specimen measured 7.0 x 5.5 x 1.4 cm and was flat and well delimited, with a smooth bronze-colored external surface. The sectioned surface was elastic and bronze-colored with a disorganized fascicular pattern. The histological examination demonstrated that the neoplasia was well delimited and made up of three characteristic components: fusi-form cells forming small fascicles, numerous blood vessels and adipose tissue interspersed between the fusiform cells. These cells had ovoid basophilic nuclei and eosinophilic cytoplasm that was clear and well defined. Dispersed cells with large pleomorphic and hyperchromatic nuclei were seen. Mitoses were scarce in number and mast cells were frequently found to permeate the tumor. The stroma was myxoid and hypocellular. There were no areas of necrosis or hemorrhage. The vessels were of small to medium caliber, with open lumens and walls that were frequently hyalinized, and passed haphazardly through the tumor.

The fusiform cells were positive for vimentin and negative for CD34, protein S-100, actin and desmin.

## DISCUSSION

**Figure 1:**
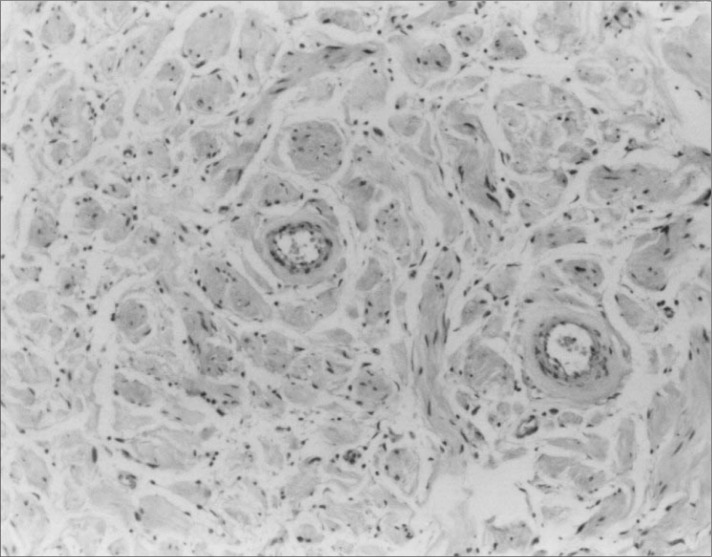
Photomicrograph of the angiofibroma showing its three characteristic components: fusiform cells, blood vessels and adipose tissue (hematoxylin-eosin; 4 X).

**Figure 2:**
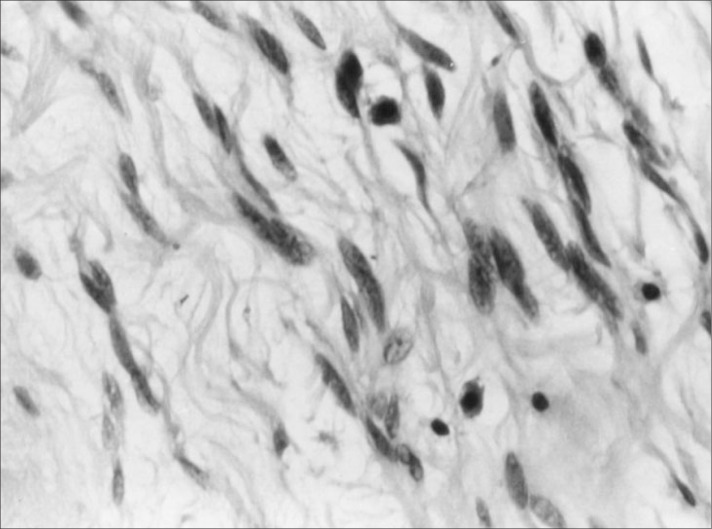
Small to medium size vessels with hyalinized walls, showing fusiform cells with bland nuclei and clear cytoplasm in an angiofibroma tumor (hematoxylin-eosin; 20 X).

Cellular angiofibroma of the vulva arises in middle-aged women as a small painless vulvar mass,^1,2^ as occurred in our case. The clinical diagnosis is usually Bartholin's glandular cyst. Histologically, these lesions are characterized by fusiform cells without atypia, forming small fascicles in the middle of collagen bundles, and frequent blood vessels of small to medium caliber, sometimes with a hyalinized wall and a variable component of mature adipocytes (Figures 1 and 2). Such tumors generally present mitotic activity and there may be sparse atypical cells in the stroma, but necrosis is absent.^1,2^

In the case described, the tumor measured 7 cm in diameter and had a very low mitotic index, which may have impeded the diagnosis. The cases previously described in the vulva measured less than 3 cm in diameter.^1^ Garijo and Val-Bernal^3^ described a very similar lesion, located in the subcutaneous layer of the thoracic wall, measuring 7 cm in diameter and without mitoses. The authors commented that the size of the lesion, the low mitotic activity and the collagen formation in the stroma might have been due to the longer development time of the lesion until its diagnosis and excision, in relation to the cases of Nucci et al.^1^

The immunohistochemical study showed positivity only for vimentin, which suggests, according to the literature, that the tumor cells are of fibroblastic origin.^1^ In 1998, Laskin et al.^4^ described 11 very similar cases of lesions in the male genital tract, which they called tumors of angiomyofibroblastoma type of the male genital tract. According to these authors, such lesions are derived from stem cells, with a capacity for adipose and myofibroblastic differentiation in accordance with the influence of hormones, microenvironments, cytokines and growth factors.

Cellular angiofibroma of the vulva appears to be benign, since there is no report of tumors that progressed with metastasis. Nonetheless, the follow-up described in most cases is short. Local excision with free margins is the appropriate treatment.^1,2^ In our case, resection of lesion was performed and the patient presented no signs of relapse four months later.

Specific tumors of soft parts of the vulva form part of the differential diagnosis, such as aggressive angiomyxoma and angiomyofibroblastoma. The first of these is an infiltrative lesion with a tendency towards recurrence that is paucicellular and has extensively myxoid stroma. Angiomyofibroblastoma is generally less cellular than cellular angiofibroma and presents the cells arranged around the small-caliber blood vessels. These two lesions are positive for desmin and/or actin.^1,2^ In fact, all three of these lesions seem to form part of a spectrum of mesenchymal lesions of the vulva with fibroblastic and myofibroblastic differentiation.^1^ Other soft-part lesions form differential diagnoses and can be ruled out in accordance with the histology and immunohistochemistry.^2^
